# Corneal Endothelial Cell Density in Normal Tension Glaucoma Compared to Healthy Controls

**DOI:** 10.3390/jcm11123515

**Published:** 2022-06-18

**Authors:** Jia Xu, Manishi A. Desai, Hyunjoo J. Lee

**Affiliations:** Department of Ophthalmology, Boston University School of Medicine, Boston Medical Center, Boston, MA 02118, USA; jeanniex@bu.edu (J.X.); manishi.desai@bmc.org (M.A.D.)

**Keywords:** corneal endothelial cell density, normal tension glaucoma, mean cell size, coefficient of variance

## Abstract

The purpose of this study was to investigate corneal endothelial cell density (ECD) and morphology between normal tension glaucoma (NTG) and controls. A cross-sectional, single center study of 24 NTG and 26 age-matched healthy eyes were included. ECD, mean cell size (MCA) and coefficient of variance (CV) were analyzed, controlling for age and number and duration of concurrent glaucoma medications. NTG subjects had significantly lower ECD (2307 ± 514.7 vs. 2558 ± 278.5, *p* = 0.044) and larger MCA (458.3 ± 94.8 vs. 386.7 ± 57.3, *p* = 0.004), but no difference in CV compared to healthy subjects. NTG subjects stratified by number of glaucoma medications showed significant differences in ECD (*p* = 0.024) and MCA (*p* = 0.021), but no difference in CV. There were no significant differences in ECD, MCA or CV between subjects stratified by duration of glaucoma medication usage. After age-adjusting, there was no dose-dependent relationship between mean ECD or MCA and number of glaucoma medications. Post hoc analysis demonstrated only NTG subjects on three or more glaucoma medications had statistically significant differences in ECD (*p* = 0.032) and MCA (*p* = 0.037) compared to NTG subjects on two glaucoma medications. This study suggests that NTG is associated with lower corneal endothelial cell density and mean cell size.

## 1. Introduction

Corneal endothelial cells (CEC) are critical to maintaining clear vision by functioning as a diffusion barrier and by performing active ion transport to regulate corneal hydration [1]. This cell layer has no known mitotic properties in humans, and instead adapts to injury by varying size, known as polymegathism, or shape, known as pleomorphism [2]. In adult eyes, the average corneal endothelial cell density (ECD) is 2000–3000 cells/mm^2^ [3]. ECD decreases 0.6% per year with concurrent increases in polymegathism and pleomorphism reflective of age-related attrition [4].

Glaucoma has been associated with decreased ECD and polymegathism [5,6,7,8]. The reason for this is not completely understood, but elevated intraocular pressure (IOP) has been associated with these CEC changes [9]. It has been proposed that damage to CECs results from mechanical forces related to elevated IOP [8]. In primary open-angle glaucoma (POAG), trabecular meshwork (TM) cellular density is reduced compared to non-glaucomatous eyes [10]. CEC and TM cells share a common embryonic progenitor [11]. CEC changes could reflect the status of TM cells, or be similarly vulnerable in glaucomatous eyes, possibly independent of IOP.

Normal tension glaucoma (NTG) is a subset of POAG in which glaucomatous optic nerve head cupping with corresponding visual field loss occurs without an elevated IOP (<21 mm Hg) [12]. ECD was significantly reduced in NTG eyes compared to POAG eyes in a study of 58 eyes in China [13]. These results raised the possibility that high IOP may not be the only reason for lower ECD or other CEC changes in glaucoma. However, another study of 227 eyes in Korea found no difference between NTG and non-glaucomatous eyes [14].

The aim of our study was to elucidate the relationship between ECD and NTG in a multi-racial population, given prior conflicting results. Additionally, we were interested in investigating ECD and CEC morphology as an additional screening parameter for NTG patients, whose normotensive IOP can lead to delayed glaucoma diagnosis until irreversible optic nerve head cupping is seen on dilated fundus examination or visual field deficits are symptomatic [15].

## 2. Materials and Methods

### 2.1. Study Design and Subjects

Patients examined in the Department of Ophthalmology at Boston Medical Center, Boston, USA between January 2016 to November 2018 were screened for enrollment. We included subjects 18 years and older, who had been given a diagnosis of NTG by an attending glaucoma specialist. If both eyes met inclusion criteria, the right eye was chosen arbitrarily for the study. Eyes with any documentation of IOP ≥ 21 mmHg were excluded in the glaucoma group. Eyes with any history of prior surgery, laser eye treatment, known ocular trauma, pre-existing corneal disease, uveitis, inflammation, or other optic atrophy were excluded from both groups. Contact lens users were not excluded.

Control subjects were age-matched in frequency to the NTG group, and included patients who visited the clinic for a comprehensive eye exam, or for a cataract evaluation, and who did not have any suspicion of glaucoma.

### 2.2. Materials

Corneal endothelial cells were imaged using a non-contact specular microscope (Tomey EM-3000, Phoenix, AZ, USA) by a single investigator (JX). The images were captured at the central cornea and assessed using the microscope’s software with automatic tracing and analysis of the corneal endothelial cells. Central corneal thickness (CCT) was also assessed using the specular microscope software. IOP measurements were recorded from the clinic visit note on the same day. Demographic data collected from each patient included patient age, gender, and race. Lastly, usage of IOP-lowering medications, including duration of treatment, class of medication, and number of different medications used were obtained from the medical record.

### 2.3. Outcomes

The primary outcome measure was the ECD. Secondary outcomes included mean corneal cell size (MCA) and coefficient of variance (CV). All NTG patients were using IOP-lowering medications. We stratified NTG subjects by duration of medication usage into 3 categories: 1 year or less, 2–4 years, and 5 years or more. We also stratified NTG subjects by current number of different IOP-lowering medications in 3 categories: 1 drop, 2 drops, and 3 or more drops. Finally, we compared NTG subjects who were on topical carbonic anhydrase inhibitors (CAIs) and NTG subjects who were not on topical CAIs due to the possibility of CAI-related corneal decompensation, especially in patients with lower endothelial cell counts [16,17].

### 2.4. Statistical Analysis

We performed a power analysis to determine that in order to detect a difference in mean ECD of 2500 versus 2250 cells/mm^2^, with a standard deviation of 300 cells/mm^2^, which is consistent with previously published differences [8,13,14], inclusion of 22 eyes in both the NTG and control groups would achieve 80% power with a *p*-value of 0.05. Differences in demographic data were analyzed using a chi-square test or a Fisher’s exact test. A non-paired two-tailed Student’s *t*-test was used to analyze differences in age, ECD, MCA, CV, IOP and CCT between NTG and age-matched controls. An analysis of variance (ANOVA) was used to test ECD, MCA, and CV differences between categories of duration of medication use, and number of medications used. Variables that were significant underwent post hoc analysis, controlling for age as a possible confounder. An analysis of covariance (ANCOVA) was used to test differences in ECD, MCA, and CV between the 3 different number of glaucoma medications used, adjusting for age as the covariate for each case. Differences in ECD and MCA between the numbers of medications categories also underwent post hoc analysis, with pairwise comparisons via least square analysis using Tukey-Kramer adjustments of each medication group. Lastly, differences between NTG patients on topical CAIs were analyzed with a non-paired two-tailed Student’s *t*-test. Significance was defined as *p* < 0.05 for all tests.

## 3. Results

A total of 24 eyes of 24 NTG patients and 26 eyes of 26 age-matched control patients were included in our analysis. Patient demographics are presented in Table 1. No significant differences were found between the distribution of age, gender, race, and eye analyzed of NTG and control subjects. The predominant represented race was African American (71% in NTG, 46% control).

Comparisons of ECD, MCA, CV, CCT, and IOP on the day of specular microscopy imaging between the NTG and control eyes are presented in Table 2. Significant differences found between the NTG and control eyes included: lower ECD, larger MCA, and lower IOP in NTG eyes. NTG eyes had 9.8% lower ECD compared to age-matched healthy eyes. In addition, NTG eyes had significantly more polymegathism, as reflected by a 18.5% higher MCA compared to healthy eyes. There were no significant differences in CV or CCT between NTG and healthy eyes. 

In order to examine the potential effect of glaucoma medications on CECs, we analyzed the differences in ECD, MCA and CV between NTG eyes stratified by number and duration of glaucoma medications (Table 3 and Table 4). There were no significant differences in age between the groups stratified by number of drops (Table 3) or by duration of glaucoma medication use (Table 4). We found a significant difference in ECD and MCA in NTG eyes on 1, 2, or ≥3 glaucoma drops (Table 3). However, these differences in ECD or MCA were not dose-dependent. The ECD for NTG eyes on 1 drop or ≥3 drops was lower than in the 2 drops group. Additionally, the MCA for NTG eyes on 1 drop or ≥3 drops was higher than in the 2 drops group. We did not find a significant difference in CV when stratifying by number of glaucoma medications. There were also no significant differences in ECD, MCA or CV between 3 groups stratified by duration of glaucoma medication use (Table 4).

After correcting for age, we again found a significant difference in ECD and MCA in NTG eyes on 1, 2, or ≥3 glaucoma drops (Table 5). Post hoc analysis showed that the only statistically significant differences in ECD and MCA between number of glaucoma medication sub-groups were between eyes on ≥3 glaucoma drops and eyes on 2 drops (Table 5), with lower ECD and higher MCA in eyes on ≥3 glaucoma drops. NTG patients on a topical CAI (dorzolamide) did not show any significant difference in ECD, MCA, or CV compared to NTG patients on non-CAI glaucoma medications (Table 6).

## 4. Discussion

In our single-center study of a predominantly African American patient population, normal tension glaucomatous eyes had significant lower corneal endothelial cell counts and more polymegathism compared to frequency-matched healthy controls. The differences in ECD and MCA between NTG eyes and control eyes were significant even after fully adjusting for age. These differences were particularly pronounced in eyes using ≥3 topical glaucoma medications. 

The theories behind the mechanism of lower CEC counts in glaucoma patients are still being debated. By analyzing NTG patients, our aim was to explore theories outside of the mechanical damage of direct compression from increased IOP. Our results lend some support to a congenital theory proposed by Gagnon et al. that glaucomatous eyes, which experience increased resistance in the trabecular meshwork and decreased TM cell density, may also show decreased CEC density and CEC morphology given that CEC and TM cells arise from a shared progenitor [8]. However, much remains to be elucidated about mechanisms of CEC loss in glaucomatous eyes, and whether CEC changes are primary or secondary findings of glaucoma.

Reduced ECD in NTG patients compared to healthy controls has been inconsistently shown in previous studies. Zarnowski and colleagues found significantly lower ECD in NTG (2342 ± 394 mm^2^ NTG vs. 2732 ± 356 mm^2^ control) patients compared to non-glaucomatous patients [7]. However, a study by Gagnon and colleagues found no significant difference in ECD between NTG (2255 ± 321 mm^2^) and non-glaucomatous eyes (2560 ± 306 mm^2^) [8]. Interestingly, in their study, the mean ECD of NTG eyes was similar to that of POAG eyes (2226 ± 311 mm^2^), which was noted to be significantly lower than non-glaucomatous eyes by a small margin. Their analysis was limited by inclusion of only five NTG patients, and of a solely all-White population.

Another study conducted by Cho and colleagues also found no significant changes in ECD in NTG patients (2696.7 ± 303.9 mm^2^) when compared to non-glaucomatous patients (2723.6 ± 300.6 mm^2^) [14]. In this study, the mean age of their NTG patients was younger than our population by about eight years, and the severity of NTG in each of their newly diagnosed subjects was not reported. Moreover, patients previously using glaucoma medications were excluded. Their results could suggest that topical glaucoma medications may be to blame for lower ECD in NTG patients, but it is also possible that more advanced NTG could also lead to lower ECD independently from eye medication use, and that by excluding patients on glaucoma drops, Cho and colleagues may also have excluded more advanced cases of NTG.

Furthermore, Lee and colleagues compared NTG to POAG (2380 ± 315.3 mm^2^ NTG vs. 2540.0 ± 320.4 mm^2^ POAG) eyes, and found significantly decreased ECD in NTG patients despite significantly higher IOP in the POAG group [13]. They conducted a univariate analysis that was not adjusted for IOP or other variables. In our study also, the significantly higher IOP in healthy eyes did not correspond to lower ECD, suggesting a mechanism of CEC loss related to either medication use, or a mechanism that is independent of IOP.

In comparing these previously published results to our own, it is important to recognize that the populations in these previous studies conducted in Poland, Canada, South Korea, and Hong Kong, respectively, were very likely quite different from our study population, which was predominantly African American. Our results help to show that decreased ECD in NTG eyes is not a phenomenon unique to certain populations, including in races with a higher prevalence of open-angle glaucoma [18].

In addition to CEC density, CEC polymegathism (variations in MCA) and pleomorphism (variations in CV) are also reflective of CEC damage. MCA was also found to be significantly different between NTG patients and non-glaucomatous patients. While most prior studies have focused on ECD, the additional finding of elevated MCA reflects the compromised CEC status and has been previously reported in other open-angle glaucomas [5]. We did not find significant differences in CV in NTG eyes and control eyes. Loss of uniformity amongst hexagonal CEC may be a sign of endothelial cell decompensation. However, CV may not be the most reliable marker because the calculation of CV is susceptible to small changes in a few areas of cells [19]. It has been shown that eyes with normal ECD may be skewed towards abnormally high CV if a small population of CEC are abnormal [20].

Additionally, CCT in NTG has been shown to be thinner [14,21,22,23] versus no different from normal eyes [13,24]. CCT is important to consider because thinner CCTs can underestimate the IOP, and if elevated IOP were simply underestimated in NTG patients, this would lend support to the mechanical damage theory of reduced ECD. However, CCT was not significantly thinner in our NTG patients compared to our controls.

Our study had a number of limitations. All of our NTG patients were on topical glaucoma therapy, which limited our ability to determine whether CEC profiles are due to primary NTG or possibly secondary medication-related changes. In vitro models have shown that various IOP-lowering drops can be cytotoxic to CECs [24]. In particular, topical CAIs are thought to affect CEC pump function, and thus could be cytotoxic to CECs, especially in eyes with low ECD and less functional reserve [17,25]. When we compared NTG eyes on topical CAIs to NTG eyes on other topical glaucoma medications, we found no significant differences in ECD, MCA, or CV. It is important to note that 5 of 7 eyes on CAIs were also on 3 or more glaucoma medications, while only 1 of 17 eyes not on CAIs was on 3 or more glaucoma medications, which limits the interpretation of the lack of significant difference between CAI and non-CAI users. Additionally, in vitro and animal studies have shown that preservatives in glaucoma medications, such as benzalkonium chloride, can damage CECs [26,27]. In order to account for the possible effect of any glaucoma medications, we stratified eyes based on medication exposure, and did not find medication dose-dependent or duration-dependent relationships. Whether the significant difference in CEC density only seen in the group of eyes on three or more drops is due to the severity of this group’s NTG, which requires more aggressive treatment, or the medications themselves, including the preservatives they contain, will require further study. However, previous studies have also not shown significant changes in ECD in eyes exposed to topical CAIs or other topical glaucoma medications [16,28,29]. A prospective study monitoring the change of CEC densities over time in a variety of NTG patients at different disease severities, especially those who are treatment-naïve, would help elucidate these findings. In addition, we had not specifically identified and excluded contact lens wearers, which could have confounded our ECD measurements. However, it is likely that no contact lens wearers were actually included in our study, as there are very few contact lens wearers within the targeted patient population in our institution.

A second limitation was our relatively small sample size, although the sample size was large enough to achieve adequate statistical power for our primary outcome analysis. We also employed a linear model that assumes normality while taking group sizes into account when computing hypothesis testing. A larger sample size may allow for multiple regression analysis, whereas our multi-categorical analysis was best suited for pair-wise analysis. Curiously, there was a higher ECD for eyes on two glaucoma drops compared to those on either fewer or more glaucoma medications, after adjusting for age. The reason for this finding is unclear, but could be related to unknown factors influencing how many eyedrops patients were prescribed, or the type and duration of drugs used. Further study with a larger sample size with treatment-naïve NTG patients could help determine if NTG eyes in earlier stages of disease have detectable CEC changes. Additionally, inclusion of higher IOP glaucomatous eyes that are matched for both age and glaucoma medication could also help to confirm if the mechanical hypothesis of CEC changes holds true.

Finally, the differences we saw in mean ECD and MCA between NTG and healthy controls’ eyes were small (251 cells/mm^2^ and 78 μm^2^, respectively), and the predictive value of these differences is unknown at this point. 

## 5. Conclusions

In conclusion, we found that the eyes of NTG patients have lower CEC density and larger CEC size (polymegathism) compared to healthy eyes, particularly if using three or more topical glaucoma medications, but not in a dose-dependent fashion. Further studies of CECs in NTG patients, including prior to medication initiation, with stratification by glaucoma severity and longitudinal data collection, may help confirm whether CEC profiles can potentially serve as independent risk factors for NTG. Our results do suggest that NTG eyes may be at increased risk of CEC attrition, whether due to primary or secondary causes, and monitoring CEC health with specular microscopy may help to guide decisions around intra-ocular surgery and other therapies in these patients.

## Figures and Tables

**Table 1:** Demographic and ophthalmic data of NTG patients and their matched controls.

Variable		NTG (n = 24)	Control (n = 26)
Age	Mean ± SD	63.8 ± 11.4	61.2 ± 11.2
	Range	41–83	39–82
	*p*-value	0.413	
Sex	Male (%)	9 (38%)	15 (58%)
	Female (%)	15 (62%)	11 (42%)
	*p*-value	0.153	
Race	African American (%)	17 (71%)	12 (46%)
	Hispanic (%)	2 (8%)	9 (35%)
	Caucasian (%)	2 (8%)	2 (8%)
	Asian (%)	3 (13%)	3 (12%)
	*p*-value	0.155	
Eye	Right Eye (%)	17 (71%)	22 (85%)
	Left Eye (%)	7 (29%)	4 (15%)
	*p*-value	0.314	

**Table 2:** Specular microscopy parameters and IOP of NTG patients and their matched controls.

		NTG (n = 24)	Control (n = 26)
ECD (cell/mm^2^)	Mean ± SD	2307 ± 514.7	2558 ± 278.5
	Range	1487–3748	2004–3023
	*p*-value	0.044 *	
MCA (μm^2^)	Mean ± SD	458.3 ± 94.8	386.7 ± 57.3
	Range	341–673	208–499
	*p*-value	0.004 *	
CV	Mean ± SD	43.8 ± 14.8	43.9 ± 6.1
	Range	29–96	33–55
	*p*-value	0.956	
CCT (μm)	Mean ± SD	506.6 ± 36.4	510.5 ± 28.0
	Range	459–574	459–582
	*p*-value	0.690	
IOP (mmHg)	Mean ± SD	13.0 ± 2.5	16.3 ± 3.1
	Range	8–18	11–20
	*p*-value	<0.001 *	

* Statistically significant (*p* < 0.05).

**Table 3:** ANOVA Models: CEC parameters stratified by number of glaucoma medications.

Number of Glaucoma Medications		1 (n = 11)	2 (n = 7)	≥3 (n = 6)
Age	Mean ± SD	59.0 ± 11.7	65.6 ± 10.2	70.5 ± 9.6
	*p*-value	0.121		
ECD (cell/mm^2^)	Mean ± SD	2347.7 ± 402.1	2616.0 ± 603.5	1872.7 ± 307.5
	*p*-value	0.024 *		
MCA (μm^2^)	Mean ± SD	440.4 ± 94.0	411.9 ± 60.6	545.2 ± 80.6
	*p*-value	0.021 *		
CV	Mean ± SD	44.5 ± 17.6	39.6 ± 5.6	47.2 ± 17.7
	*p*-value	0.655		

* Statistically significant (*p* < 0.05).

**Table 4:** ANOVA Models: CEC parameters stratified by years of glaucoma medication use.

Years of Medication Use		≤1 (n = 7)	1–5 (n = 8)	≥5 (n = 9)
Age	Mean ± SD	62.7 ± 12.4	62.8 ± 12.5	65.6 ± 10.9
	*p*-value	0.854		
ECD (cell/mm^2^)	Mean ± SD	2255.0 ± 489.9	2470.0 ± 650.4	2203.1 ± 412.9
	*p*-value	0.559		
MCA (μm^2^)	Mean ± SD	463.9 ± 112.3	440.9 ± 91.0	469.3 ± 93.1
	*p*-value	0.826		
CV	Mean ± SD	42.6 ± 6.0	46.1 ± 20.7	42.6 ± 14.8
	*p*-value	0.868		

**Table 5:** (**a**) ANCOVA Models: ECD and MCA stratified by number of glaucoma medications adjusting for age. (**b**) *p*-values of least squares of ECD means for number of glaucoma medications. (**c**) *p*-values of least squares of MCA means for number of glaucoma medications.

(**a**)				
**Number of Glaucoma Medications**		**1 (n = 11)**	**2 (n = 7)**	**≥3 (n = 6)**
ECD (cell/mm^2^)	Adjusted Mean ± SE	2312 ± 144.1	2629 ± 172.8	1921 ± 195.8
	Pr > F-value	0.040 *		
MCA (μm^2^)	Adjusted Mean ± SE	451.5 ± 25.5	407.7 ± 30.6	529.6 ± 34.6
	Pr > F-value	0.046 *		
CV	Adjusted Mean ± SE	44.5 ± 17.6	39.7 ± 5.6	47.2 ± 17.7
	Pr > F-value	0.488		
(**b**)				
**Number of Glaucoma Medications**		**1 (n = 11)**	**2 (n = 7)**	**≥3 (n = 6)**
1 (n = 11)			0.367	0.295
2 (n = 7)		0.367		0.032 *
≥3 (n = 6)		0.295	0.032 *	
(**c**)				
**Number of Glaucoma Medications**		**1 (n = 11)**	**2 (n = 7)**	**≥3 (n = 6)**
1 (n = 11)			0.536	0.216
2 (n = 7)		0.536		0.037 *
≥3 (n = 6)		0.216	0.037 *	

* Statistically significant (*p* < 0.05).

**Table 6:** Usage of carbonic anhydrase inhibitors within NTG patients.

		No CAIs (n = 17, ≥3 IOP meds = 1)	CAIs (n = 7, ≥3 IOP meds = 5)
ECD (cell/mm^2^)	Mean ± SD	2394 ± 528.0	2095 ± 445.2
	Range	1487–3748	1625–2935
	*p*-value	0.180	
MCA (μm^2^)	Mean ± SD	443.6 ± 95.0	493.9 ± 91.1
	Range	355–673	341–612
	*p*-value	0.249	
CV	Mean ± SD	42.6 ± 14.5	46.6 ± 16.4
	Range	33–96	29–80
	*p*-value	0.587	

## Data Availability

Not applicable.
